# Trace elements concentrations in soil contaminate corn in the vicinity of a cement-manufacturing plant: potential health implications

**DOI:** 10.1038/s41370-023-00548-8

**Published:** 2023-06-14

**Authors:** Sa’adatu Abatemi-Usman, Olubunmi Akindele, Ayansina Ayanlade, Magali Perez, Isma’il Attahiru, Gareth Norton, Joerg Feldmann, Eva Krupp

**Affiliations:** 1 Trace Element Speciation Laboratory Aberdeen (TESLA), Department of Chemistry, School of Natural and Computing Sciences, University of Aberdeen, Aberdeen, UK; 2 National Oil Spill Detection and Response Agency, Central Business District, Abuja, Nigeria; 3 MetaResponse International Limited, Abuja, Federal Capital Territory Nigeria; 4 Department of Geography, Obafemi Awolowo University, Ile-Ife, Nigeria; 5 Institute of Biological and Environmental Sciences, University of Aberdeen, Aberdeen, UK; 6 Trace Element Speciation Laboratory (TESLA), Institute for Chemistry, University of Graz, Graz, Austria

**Keywords:** Metals, Farmland, Human health, Exposure, Hazard impact, Nigeria

## Abstract

**Background:**

Cultivated lands in the vicinity of industry are vulnerable due to trace element releases from industrial activities. One such situation concerns the surrounding of the largest cement-manufacturing plant in sub-Saharan Africa, located in Obajana, Nigeria.

**Objective:**

This study aimed at examining the trace element concentrations in the soil as they contaminate corn crops in the vicinity of a cement manufacturing plant. A case study of the cement-manufacturing plant located in Obajana, Nigeria is presented.

**Methods:**

We used inductively coupled plasma-mass spectrometer to analyse for total arsenic (As), cadmium (Cd), chromium(Cr), copper (Cu), lead (Pb), and nickel (Ni) concentrations and microwave-induced plasma-atomic emission spectrometer to measure total iron (Fe) and zinc (Zn) contents in 89 samples of corn and surface soil (0–15 cm) from five farmlands including reference farmland and evaluated health hazard of human exposure to the trace elements via the consumption of corn cultivated in the area.

**Results:**

The results showed the average Cr concentrations in µg/g dry weight (±standard error of the mean) in corn ranged from 2.08 ± 0.17 to 3.56 ± 0.65 in all the farmlands including control, while the mean Pb levels in µg/g dry weight (± standard error of the mean) in corn extended from 0.23 ± 0.03 to 0.38 ± 0.02 in the farmlands downwind of the cement plant. The Cr values were several factors higher than the stable concentration range of 0.01 to 0.41 µg/g reported in cereal grains, while the Pb values exceeded the limit of 0.2 µg/g set by the Food and Agriculture Organization of the United Nations/World Health Organization in grains. Lead is a trace element of environmental concern and its average levels in the farmlands downwind of the plant were found to be several orders of magnitude higher than the values in µg/g dry weight (± standard error of the mean) (0.01 ± 0.00 to 0.02 ± 0.00) observed in the farmlands upwind of the plant and were statistically significant (*p* < 0.0001).

**Impact statement:**

Our findings provide the first health hazard assessment from the consumption of corn cultivated in the vicinity of the largest cement-manufacturing plant in Nigeria as far as we know.

## Introduction

The chemical constituents such as trace elements of the surrounding environment like soil and food determine the quality of life of human beings [1, 2]. Soil acts as a sink and has the potential for contaminants’ transference to the food chain through crop production [3]. Soil quality, particularly its contamination level is closely linked to human well-being [4, 5], as many food commodities can accumulate trace and toxic elements [6–8] which often induce cancer. Consequently, human health could be impacted by certain critical levels of trace elements through crop plant consumption [9]. For example, critical Cd contents in plants which are above the level regarded as desirable for humans are 5 to 30 mg/kg [9]. Some studies have reported that both natural and human-induced contamination impact the trace element contents of cultivated soils and food crops like corn and this results in an assortment of both essential and toxic elements in such crops [9–15]. The foremost threats to human well-being from trace elements, for example, are linked with exposure to arsenic (As), cadmium (Cd), lead (Pb), mercury (Hg) [11], as well as to chromium (Cr) and nickel (Ni) which therefore makes these elements of environmental importance. Distinct exposure-response associations and high risks have been noted with ingestion [11]. The consumption of Cd-contaminated food could cause kidney dysfunction, hypertension, and bone fractures [10], while elevated Cr levels could lead to enhanced red blood cells production and an abnormality of the thyroid artery [13]. Similarly, Ni intake may be associated with some health effects such as fatigue, cardiac arrest, and respiratory problems [12], while excessive consumption of Pb is linked to memory declination and an imbalance in the manifested behaviour in children [13], nervous, cardiovascular and bone diseases in adults [16] as well as anaemia. An excess of zinc (Zn) in the body is known to lead to sideroblastic anaemia [12]. Therefore, cultivated plants represent a major pathway for the movement of potentially toxic trace elements from soils to humans [10, 12]. Information on the bioaccumulation of trace elements in animals and humans has been growing and has highlighted the importance of naturally occurring trace elements in soils [6, 17], although human-induced enrichment is also a major aspect [17]. Many trace elements are essential to both humans and plants. However, the toxicity levels of some of these elements are lower in humans and animals than in plants. Various strategies and methods, including assessments of dietary exposure have been recommended for human health hazard evaluation and for safe food characterisation [18]. Therefore, many countries have undertaken trace element monitoring in foods [2]. As children are more sensitive to contaminants than adults, health hazard investigation need to be considered separately as the contact pathway through food varies with age [19].

At present, the processes used for cement production could lead to trace element emissions into the environment [20]. Such emissions are also linked with cement transportation [21], as a kilogram of cement production could generate about 0.07 kilograms of dust in the atmosphere daily [22]. Seventeen trace elements can be found in cement dust: full suite includes As, antimony (Sb), beryllium (Be), Cd, Cr, Cu, cobalt (Co), Hg, manganese (Mn), Ni, Pb, selenium (Se), tellurium (Te), thallium (Tl), tin (Sn), vanadium (V) and Zn [20]. The spread of cement dust through rain and wind could cover a large area and become integrated into soils and plants, and subsequently humans in a food chain effect [21].

In this study, we collected corn (*Zea mays*) grains and surface soil (0–15 cm) samples from four farmlands in the vicinity of a major cement-manufacturing plant in Obajana, Kogi State in north-central Nigeria and from a fifth farmland that served as a reference site. Total As, Cd, Cr, Cu, Fe, Ni, Pb and Zn concentrations were determined in all the samples. Corn was selected as it is a staple food for people with different socio-economic backgrounds and diet preferences in Nigeria [23–25]. In addition, *Zea mays* is among the few plants which accumulate larger amounts of Pb, even though it is only slightly available to plants in most soils [2], and Zhang, et al. [26] had found it to be the main source of Pb consumption through food among the common cereals including foxtail millet, rice, and wheat which are all staples in Nigeria. Our theory (put to test) was that farmlands with corn crops cultivated in the vicinity of the largest cement-manufacturing plant in Obajana, Sub-Saharan Africa (SSA), with decades-long production, could present a significant health hazard to humans from potentially toxic trace elements like Pb especially due to increased vehicular traffic and prevailing wind direction. Therefore, the aim of this study was to identify the degree of contamination and the objectives were: (i) to assess the effect of Obajana cement plant (OCP) and its associated activities such as the movement of products on trace element depositions in surface soil and *Zea mays*, and (ii) to evaluate the non-carcinogenic health hazard impact to consumers of the corn because of any depositions. Evaluating the trace element contents in corn could serve as an indicator of changes in the terrestrial environment in the vicinity of OCP. Our findings could make a statement on food safety and aid policy releases on farmlands’ proximity to industrial areas in Nigeria.

## Materials and methods

### Location description

**Fig. 1:**
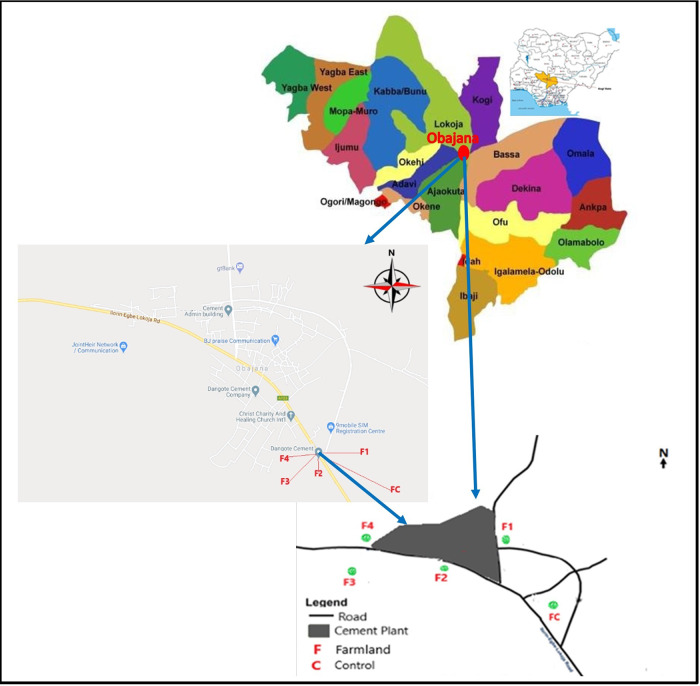
Map of the study area in Obajana, Kogi State, Nigeria, and the locations of the sampling sites in relation to Obajana cement plant.

The study sites are in Obajana, Kogi State in north-central Nigeria (Fig. 1), in the vicinity of OCP and a reference site. Obajana cement plant, the largest cement-manufacturing plant in SSA has been in operation since 1992 and has an annual capacity of about 16.3 million tonnes of cement, as at 2023. Obajana town has the geographic coordinates of 7° 55′ 0″ North and 6° 26′ 0″ East and is 205 m above sea level. The area has a tropical savanna climate with a constant annual temperature of over 18 °C. There are distinct rainy and dry seasons spanning from April through September and from December through March respectively. The soil type in the area is a mixture of Nitisol (a well-drained soil with 30% clay) and Lixisol (Sonneveld, 1998), and comprises relatively high organic carbon (C, 1.4–2%), high potassium (K, 0.61–0.73 kmol/kg), moderately low nitrogen (N, 0.11–0.15%), moderate phosphorus (P, 7–20 µg/g), moderate calcium (Ca, 2–5 µg/g), moderate magnesium (Mg, 1–2.5 µg/g) and moderate Zn (1–5 µg/g) (Sonneveld, 1998). North-easterly winds predominate in the region. The communities are located to the west and south next to the site of OCP. The occupation of the inhabitants of Obajana includes rain-fed farming, petty trading, hunting, and cattle rearing. Cottage farming is the farming system found in the area, with farmland sizes between 0.5 and 2 hectares (ha). The major cultivated crops in the locality include corn, pepper, rice, cassava, millet, cowpea, and sweet potatoes.

### Study design and sampling approach

**Fig. 2:**
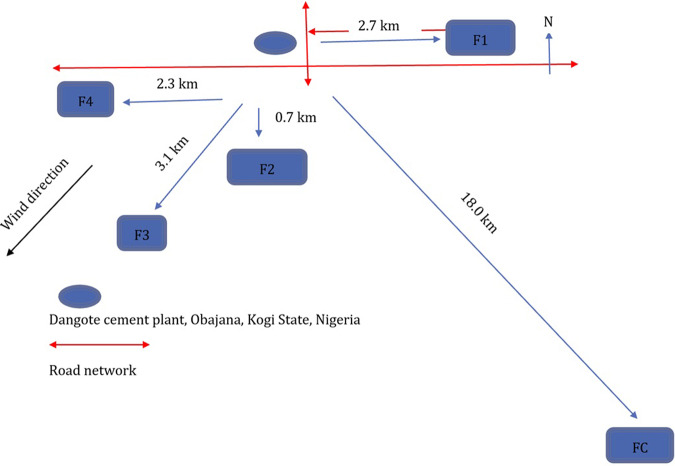
Schematic of the sampling sites in relation to Obajana cement plant (OCP) and the sites’ sizes in relation to each other regarding the sampling regime.

The study design recognised both wind direction which was measured using a wind vane in the study area, as well as the proximity of the cultivated farmlands to OCP regarding vehicular traffic (Fig. 2). Four farmlands, designated as F1–F4 and of mix Nitisol and Lixisol soil types were selected in the immediate environment of OCP (Figs. 1 and 2). Farmland 1 (F1) was located upwind of OCP at 2.7 km east (E) of the plant, while farmlands 2–4 (F2–F4) were located downwind of the plant at the following distances: F2, at 0.7 km south (S) of OCP, F3, at 3.1 km south-west (SW) of OCP, and F4, at 2.3 km west (W) of OCP respectively. A reference farmland, designated as control farmland (FC) and of similar mix Nitisol and Lixisol soil type as F1-F4 was selected upwind of OCP at 18.0 km southeast (SE) of the plant (Figs. 1 and 2). Consideration was given to getting representative samples from field sites regarding the approximate sizes of the farmlands to compare them (Table 1). Therefore, 19, 20, 24, 12 and 14 *Zea mays* cobs and surface soil (0–15 cm) from the sampled plants’ root zone were collected from FC, and F1–F4 during the harvesting period in 2017. No subsamples were made. Corn on the cob was collected after the grains had matured at four months post-cultivation from a random selection of plants. A plastic scoop was used to collect the soil from the surface layer after the whole plant was pulled out. A ruler was put in place to ensure that soil samples were within 15 cm depth. A scalpel was used to separate the grains from the cobs. Corn and soil samples were bagged in Ziploc bags and stored at −4 °C for onward transportation to the University of Aberdeen for analysis of individual samples (phytosanitary certificate number NAQS/FT/2017/001073). Milli-q de-ionised water (>18 MΩ cm, Millipore, Bedford MA, USA) was used in all investigations. HNO_3_ (Fisher Scientific, UK) and H_2_O_2_ (Sigma Aldrich, UK) used for all sample extractions were of analytical grade at 69% and 32% respectively. A multi-element stock solution (SPEX, UK) was diluted serially in milli-q de-ionised water and acidified with HNO_3_ to 10% to serve as the working calibration standards.

**Table 1:** Description of farmlands and samples collected.

Farmland	Location Lat/Long (GPS data of the corners)	Size in hectares	Number of samples	
			Surface soil	Corn
Control (C)	❖ N07.85211 ^o^ / E006.54504 ^o^	1.3	19	19
	❖ N07.85175 ^o^ / E006.54437 ^o^			
	❖ N07.85221 ^o^ / E006.54359 ^o^			
	❖ N07.85287 ^o^ / E006.54396 ^o^			
One (1)	❖ N07.92461 ^o^ / E006.43878 ^o^	1.5	20	20
	❖ N07.92425 ^o^ / E006.43957 ^o^			
	❖ N07.92477 ^o^ / E006.43968 ^o^			
	❖ N07.92489 ^o^ / E006.43890 ^o^			
Two (2)	❖ N07.92633 ^o^ / E006.40979 ^o^	1.7	24	24
	❖ N07.92596 ^o^ / E006.40993 ^o^			
	❖ N07.92629 ^o^ / E006.41138 ^o^			
	❖ N07.92615 ^o^ / E006.41129 ^o^			
Three (3)	❖ N07.92479 ^o^ / E006.39993 ^o^	0.9	12	12
	❖ N07.92467 ^o^ / E006.39976 ^o^			
	❖ N07.92439 ^o^ / E006.40004 ^o^			
	❖ N07.92456 ^o^ / E006.40019 ^o^			
Four (4)	❖ N07.92589 ^o^ / E006.40088 ^o^	1.6	14	14
	❖ N07.92557 ^o^ / E006.40048 ^o^			
	❖ N07.92583 ^o^ / E006.40016 ^o^			
	❖ N07.92609 ^o^ / E006.40047 ^o^			

### Sample preparation and quality assurance

HNO_3_ (2.5 mL, 69%) was added to ~0.1 g of soil sample (oven dried, sieved to <2 mm and ball-milled) and left overnight before adding H_2_O_2_ (2.5 mL, 32%). The mixture was heated slowly on a block digester from room temperature to 100 °C for 1 h and reheated for 1 h at 120 °C. After cooling to room temperature, more H_2_O_2_ (1 mL, 32%) was added and the new mixture was heated at 140 °C for 1 h. Extracts were then centrifuged at 3500 rpm for 5 min (ALC 4218 centrifuge, ALC International S.R.L., Italy) and 3 mL of the supernatant was diluted to 30% in the measurement vial by adding milli-q de-ionised water to the 10 mL mark before analysis. Inductively coupled plasma-mass spectrometer (ICP-MS, Model 7900, Agilent, USA) was used to measure total As, Cr, Cd, Cu, Ni and Pb concentrations. Due to issues of interference with the ICP-MS measurement, microwave-induced plasma-atomic emission spectrometer (MP-AES, Model 4100, Agilent, USA) was used to measure total Fe and Zn contents.

For corn preparation, HNO_3_ (2 mL, 69%) was added to ~0.1 g of corn (oven dried and ground to flour) (Coffee grinder, Krups F203, Germany) and left overnight. H_2_O_2_ (2 mL, 32%) was then added and the mixture was microwave extracted (MARS 5, CEM Corporation, USA) for 5 min at 50 °C, 5 min at 75 °C and 25 min at 95 °C. Centrifugation and measurement of metals in sample were as defined in the preceding section. The analytical procedure was validated using certified reference materials (CRMs). Corn meal reference material CRM-CM-A (High Purity Standards, UK) and soil reference material NIST 2709 and NIST 2711 (National Institute of Standards & Technology, USA) were prepared and analysed alongside the samples for quality assurance for total elemental analysis. The mean percent recovery ranged between 78 and 120% for the elements of interest (Table 2). Blank determinations of the analytes were also done to ensure the reliability of the measurement.

**Table 2:** Trace element concentration and percent recovery in certified reference materials (mean ± SD; µg/g).

Element	Certified reference materials								
	CRM-CM-A			NIST 2709			NIST 2711		
	Certified value	Measured value	Percent recovery	Certified value	Measured value	Percent recovery	Certified value	Measured value	Percent recovery
Arsenic	<0.01	<0.01	100	-	-	-	-	-	-
Cadmium	<0.01	<0.01	100	0.38 ± 0.01	0.46 ± 0.01	120	-	-	-
Chromium	-	-	-	130 ± 4	104 ± 2	80.0	47	39	83
Copper	2.0 ± 0.2	1.8 ± 0.0	90	34.6 ± 0.7	34.0 ± 0.1	98.2	-	-	-
Iron	-	-	-	-	-	-	-	-	-
Lead	<0.01	<0.01	100	18.9 ± 0.5	14.8 ± 0.1	78.2	1162 ± 31	1312 ± 24	113
Nickel	<0.05	<0.05	100	88 ± 5	89 ± 4	101	20.6 ± 1.1	21.1 ± 1.2	102
Zinc	21 ± 2	22 ± 3	105	-	-	-	-	-	-

### Data analysis

The ratio of the element concentration in corn (*C*_corn_) in µg/g dry weight (dwt) to that in surface soil (*C*_surface soil_) in µg/g dwt was calculated to give the surface soil to corn transfer factor (TF) [27]. The equation for the calculation is as follows (Eq. 1):

1 $${{{{{{{\mathrm{TF}}}}}}}} = {{{{{{{\mathrm{C}}}}}}}}_{{{{{{{{\mathrm{corn}}}}}}}}}/{{{{{{{\mathrm{C}}}}}}}}_{{{{{{{{\mathrm{surface}}}}}}}}\,{{{{{{{\mathrm{soil}}}}}}}}}$$

The daily element intake for adults (DEI_A_) and children (DEI_C_) in µg/person/day/kg body weight (bwt)) related to corn consumption [3] was determined by the following formula (Eq. 2):

2 $${{{{{{{\mathrm{DEI}}}}}}}}_{{{{{{{\mathrm{A}}}}}}}}/{{{{{{{\mathrm{DEI}}}}}}}}_{{{{{{{\mathrm{C}}}}}}}} = {{{{{{{\mathrm{DCIR}}}}}}}}\,{{{{{{{\mathrm{x}}}}}}}}\,{{{{{{{\mathrm{C}}}}}}}}_{{{{{{{{\mathrm{corn}}}}}}}}}/{{{{{{{\mathrm{bwt}}}}}}}}_{{{{{{{{\mathrm{mean}}}}}}}}}$$

where DCIR is the daily corn intake rate in Nigeria at 60 g/person/day [28] and bwt_mean_ is the mean bwt, considered to be ~61 kg for adults in Africa [29]. An average bwt of 32.7 kg for children [3, 19] was used since there is paucity of data on the average bwt for children in Nigeria and Africa.

The corn-related health hazard indices for adults (HHI_A_) and children (HHI_C_) [3] was determined as a ratio of DEI_A_/DEI_C_ to the United States Environmental Protection Agency’s (USEPA’s) oral reference dose (RfD_0_) of all the measured elements (same units as DEI_A_/DEI_C_) (USEPA, 1995; [17] with the following formulae (Eqs. 3 and 4):

3 $${{{{{{{\mathrm{HHI}}}}}}}}_{{{{{{{\mathrm{A}}}}}}}} = {{{{{{{\mathrm{DEI}}}}}}}}_{{{{{{{\mathrm{A}}}}}}}}/{{{{{{{\mathrm{RfD}}}}}}}}_0$$

4 $${{{{{{{\mathrm{HHI}}}}}}}}_{{{{{{{\mathrm{C}}}}}}}} = {{{{{{{\mathrm{DEI}}}}}}}}_{{{{{{{\mathrm{C}}}}}}}}/{{{{{{{\mathrm{RfD}}}}}}}}_0$$

Human health hazards are possible outside the RfD_0_ dosage. A HHI value of less than one (<1) would imply no risk from the consumption of corn cultivated in the sampled farmlands, while HHI value of greater than one (>1) would imply a significant risk from the cultivated corn ingestion.

Minitab 19 (Minitab Inc, USA) was used to determine the descriptive statistics on a dry weight basis and to graphically analyse the results. Samples with values that were less than the detection limit of the analytical procedure were reported as less than (<) 0.001. One-way analysis of variance was used to investigate the significant differences of the mean element concentrations in the sample sets, while Tukey honestly significant tests (HSD) were carried out on statistically significant results (*p* < 0.05). Principal component analysis (PCA) and HSD were used to determine how the average trace element levels varied between farmlands.

## Results

### Concentration of elements in surface soil and corn

The average element concentrations in µg/g dwt (±standard error of the mean) and their ranges as found in surface soil (0–15 cm) and *Zea mays* samples are shown in Tables 3 and 4. Arsenic, Cr and Ni were below their detection limits in the same surface soil samples collected from F2 and F3, while Zn was not detected (limit of detection (LOD) = 0.150 ng/g) in most of the surface soil samples from all the farmlands including control. Cadmium was not detected (LOD = 0.043 ng/g) in most of the surface soils collected from F2-F4 downwind of OCP as well as in most of the corn samples from all the farmlands including control (LOD = 0.001 ng/g). Tukey pairwise comparisons showed that the average Cr and Pb concentrations in surface soil samples, and the mean levels of Zn in corn samples from FC and F1 upwind of OCP were significantly higher (*p* < 0.0001) than the average concentrations determined in surface soils (Cr and Pb only) and *Zea mays* (Zn only) from F2–F4 downwind of OCP. Conversely, the mean concentrations of Pb in corn from the farmlands upwind of OCP were significantly lower (*p* < 0.0001) than the average corn Pb contents from the farmlands downwind of OCP.

**Table 3:** Mean element concentration in surface soil (µg/g dwt ±SEM).

Element	Farmlands				
	FC (*N* = 19)	F1 (*N* = 20)	F2 (*N* = 24)	F3 (*N* = 12)	F4 (*N* = 14)
Arsenic	0.49 ± 0.04 (0.43) 0.30–1.02	0.81 ± 0.05 (0.77) 0.43–1.27	0.46 ± 0.10 (0.28) <0.01–1.49	0.24 ± 0.06 (0.37) <0.01–0.60	0.22 ± 0.01 (0.22) 0.15–0.32
Cadmium	0.05 ± 0.00 (0.05) 0.04–0.07	0.11 ± 0.04 (0.05) 0.04–0.89	<0.01 ± 0.00 (<0.01) <0.01–0.02	0.02 ± 0.02 (<0.01) <0.01–0.21	<0.01 ± 0.00 (<0.01) <0.01–0.03
Chromium	98.4 ± 13.1 (85.7) 27.0–229	104 ± 19 (55) 34.5–307	29.3 ± 7.1 (13.5) <0.01–117	15.9 ± 4.2 (21.0) <0.01–33.8	43.4 ± 3.5 (42.0) 25.8–79.0
Copper	4.14 ± 0.17 (4.05) 3.01–5.44	4.18 ± 0.29 (3.69) 2.14–6.79	4.32 ± 0.95 (1.90) 0.27–15.0	2.60 ± 0.65 (2.86) 0.27–6.13	2.04 ± 0.12 (1.96) 1.43–2.92
Iron	15350 ± 1376 (12501) 6544–30127	7697 ± 790 (6535) 198–14565	13894 ± 795 (13234) 6753–22321	6970 ± 369 (6657) 4935–9686	2927 ± 129 (2783) 2286–4122
Lead	6.64 ± 0.42 (5.85) 4.82–10.0	5.15 ± 0.32 (4.96) 2.95–7.93	3.46 ± 0.67 (1.94) 0.47–9.29	3.23 ± 0.74 (3.86) 0.47–6.72	4.00 ± 0.17 (4.11) 3.04–4.79
Nickel	9.66 ± 1.80 (7.09) 4.17–37.1	6.67 ± 1.18 (4.88) 2.20–22.1	11.9 ± 4.0 (2.98) (<0.01–90.6)	5.12 ± 1.54 (5.12) <0.01–15.6	3.09 ± 0.44 (2.48) 1.67–7.16
Zinc	1.56 ± 1.08 (<0.01) <0.01–16.3	0.47 ± 0.30 (<0.01) <0.01–5.20	3.31 ± 1.36 (<0.01) <0.01–27.5	BDL	0.89 ± 0.50 (<0.01) <0.01–5.97

Median (in parenthesis) and range of values also shown.

*SEM* standard error of the mean, *BDL* below detection limit (Zn LOD = 0.15 ng/g), *N* number of replicate samples.

**Table 4:** Mean element concentration in corn (µg/g dwt ± SEM).

Element	Farmlands				
	FC (*N* = 19)	F1 (*N* = 20)	F2 (*N* = 24)	F3 (*N* = 12)	F4 (*N* = 14)
Arsenic	0.01 ± 0.00 (0.01) <0.01–0.03	0.01 ± 0.00 (<0.01) <0.01–0.04	<0.01 ± 0.00 (<0.01) <0.01–0.03	<0.01 ± 0.00 (<0.01) <0.01–0.01	0.01 ± 0.00 (<0.01) <0.01–0.03
Cadmium	<0.01 ± 0.00 (<0.01) <0.01–0.01	<0.01 ± 0.00 (<0.01) <0.01–0.01	<0.01 ± 0.00 (<0.01) <0.01–0.03	BDL	<0.01 ± 0.00 (<0.01) <0.01–<0.01
Chromium	3.56 ± 0.65 (2.58) 1.98–14.3	2.31 ± 0.07 (2.23) 1.85–2.99	2.20 ± 0.18 (2.05) 1.63–6.14	2.08 ± 0.17 (1.82) 1.75–3.57	2.57 ± 0.17 (2.41) 1.83–3.95
Copper	3.13 ± 0.48 (2.76) 1.51–11.3	2.36 ± 0.16 (2.37) 1.15–3.85	2.21 ± 0.18 (2.04) 1.00–5.44	2.79 ± 0.25 (2.71) 1.53–4.26	2.27 ± 0.18 (2.36) 1.16–3.45
Iron	42.0 ± 3.1 (40.8) 24.3–86.9	40.0 ± 3.7 (32.7) 25.8–84.8	35.2 ± 3.6 (28.4) 21.2–99.1	41.1 ± 4.4 (36.1) 26.4–77.7	44.3 ± 6.4 (35.6) 25.3–118
Lead	0.02 ± 0.00 (0.02) 0.01–0.09	0.01 ± 0.00 (0.01) <0.01–0.05	0.23 ± 0.03 (0.29) <0.01–0.36	0.30 ± 0.03 (0.26) 0.25–0.51	0.38 ± 0.02 (0.36) 0.29–0.60
Nickel	2.10 ± 0.28 (1.76) 1.27–6.64	1.45 ± 0.05 (1.38) 1.12–1.86	1.46 ± 0.08 (1.50) 1.06–3.05	1.33 ± 0.11 (1.14) 1.10–2.28	1.70 ± 0.11 (1.59) 1.30–2.69
Zinc	28.4 ± 1.2 (29.0) 20.5–40.0	23.9 ± 1.1 (24.4) 14.1–33.5	13.5 ± 1.3 (14.6) <0.01–23.8	6.32 ± 1.11 (5.80) <0.01–13.7	18.2 ± 2.1 (16.6) 9.08–31.4

Median (in parenthesis) and range of values also shown.

*SEM* standard error of the mean, *BDL* below detection limit (Cd LOD = < 0.01 ng/g), *N* number of replicate samples.

The values for the relative standard error (RSE) calculated for all the analytes of interest in surface soil (Table 3) were higher than the RSE values calculated in corn (Table 4).

### Transfer factor and health hazard indices evaluation

The average TF values from surface soil to corn and their ranges for the determined trace elements are shown in Table 5. The mean TF values for Pb in corn were several orders of magnitude higher in F2–F4 located downwind of OCP than the values calculated in FC and F1 upwind of the plant. A similar trend was observed in the average element concentrations in corn samples from all the farmlands including the control. The average TF values for Cu in *Zea mays* were also higher in F2–F4 than in FC and F1. The control farmland, F1 and F2 recorded the highest TF values for Cd, Zn and Pb. Farmland 3 recorded the greatest values for Cr and Cu, while the highest values for As, Fe and Ni were calculated from F4.

**Table 5:** Transfer factor from surface soil to corn (TF ± SEM) and the range of values.

Element	Transfer Factor				
	FC (*N* = 19)	F1 (*N* = 20)	F2 (*N* = 24)	F3 (*N* = 12)	F4 (*N* = 14)
Arsenic	0.02 ± 0.00 (0.01–0.09)	0.01 ± 0.00 (<0.01–0.07)	0.01 ± 0.00 (<0.01–0.01)	0.01 ± 0.00 (<0.01–0.01)	0.03 ± 0.01 (0.01–0.17)
Cadmium	0.07 ± 0.2 (<0.01–0.23)	0.02 ± 0.01 (<0.01–0.09)	<0.01 ± 0.00 NA	<0.01 ± 0.00 NA	<0.01 ± 0.00 NA
Chromium	0.05 ± 0.01 (0.01–0.24)	0.04 ± 0.00 (0.01–0.07)	0.05 ± 0.01 (<0.01–0.10)	0.05 ± 0.01 (<0.01–0.10)	0.07 ± 0.01 (0.03–0.13)
Copper	0.80 ± 0.15 (0.28–3.39)	0.62 ± 0.06 (0.19–1.21)	3.74 ± 0.76 (0.17–10.6)	5.02 ± 1.63 (0.32–15.4)	1.17 ± 0.12 (0.52–2.23)
Iron	<0.01 ± 0.00 (<0.01–0.01)	0.01 ± 0.01 (<0.01–0.15)	<0.01 ± 0.00 (<0.01–0.01)	0.01 ± 0.00 (<0.01–0.01)	0.02 ± 0.00 (0.01–0.05)
Lead	<0.01 ± 0.00 (<0.01–0.01)	<0.01 ± 0.00 (<0.01–0.01)	0.34 ± 0.07 (<0.01–0.76)	0.27 ± 0.07 (0.04–0.65)	0.10 ± 0.01 (0.07–0.17)
Nickel	0.26 ± 0.03 (0.09–0.59)	0.30 ± 0.03 (0.06–0.60)	0.09 ± 0.01 (<0.01–0.20)	0.19 ± 0.03 (<0.01–0.30)	0.67 ± 0.09 (0.20–1.33)
Zinc	1.77 ± 0.06 (<0.01–1.83)	67.3 ± 55.1 (<0.01–232)	3.61 ± 1.41 (<0.01–13.4)	NA NA	4.50 ± 0.95 (<0.01–5.63)

*SEM* standard error of the mean, *NA* not applicable, *N* number of replicate samples.

The HHI_A_ and HHI_C_ values were all less than 1 (<1) and are shown in Tables 6 and 7. The control farmland showed the highest HHI_A_ and HHI_C_ values for As, Cu, Ni and Zn, while F4 showed the greatest HHI values for Pb (adults only) and Fe (adults and children). Lead HHI_A_ and HHI_C_ values were between six factors and an order of magnitude greater in F2–F4 downwind of OCP than shown in FC and F1 upwind of OCP. However, all values were less than 1 (<1). The DEI_A_ and DEI_C_ values for Cr, Cu, Fe, Ni and Zn from all the farmlands were greater than one. In all the farmlands including control, Fe showed the highest DEI_A_ and DEI_C_ values followed by Zn (Tables 6 and 7).

**Table 6:** Mean health hazard indices for trace elements in adults.

Element	RfD_0_	FC		F1		F2		F3		F4	
		DEI_A_	HHI_A_	DEI_A_	HHI_A_	DEI_A_	HHI_A_	DEI_A_	HHI_A_	DEI_A_	HHI_A_
Arsenic	0.3	0.01	0.03	0.01	0.02	<0.01	0.01	<0.01	0.01	0.01	0.02
Cadmium	1	<0.01	<0.01	<0.01	<0.01	<0.01	<0.01	NA	NA	<0.01	<0.01
Chromium	1500	3.50	<0.01	2.27	<0.01	2.17	<0.01	2.05	<0.01	2.53	<0.01
Copper	40	3.07	0.08	2.32	0.06	2.18	0.05	2.74	0.07	2.24	0.06
Iron	700	41.3	0.06	39.4	0.06	34.6	0.05	40.4	0.06	43.5	0.06
Lead	3.5	0.02	0.01	0.01	<0.01	0.22	0.06	0.29	0.08	0.37	0.11
Nickel	20	2.06	0.10	1.43	0.07	1.43	0.07	1.31	0.07	1.67	0.08
Zinc	300	27.9	0.09	23.5	0.08	13.2	0.04	6.22	0.02	17.9	0.06

*HHI*_*A*_ health hazard index for adults, *DEI*_*A*_ average daily element intake from surface soil through corn for adults, *RfD*_*0*_ reference dose of the element. DEI_A_ and RfD_0_ in µg/person/day/kg bwt, *NA* Not applicable.

**Table 7:** Mean health hazard indices for trace elements in children.

Element	RfD_0_	FC		F1		F2		F3		F4	
		DEI_C_	HHI_C_	DEI_C_	HHI_C_	DEI_C_	HHI_C_	DEI_C_	HHI_C_	DEI_C_	HHI_C_
Arsenic	0.3	0.02	0.06	0.01	0.04	0.01	0.03	<0.01	0.01	0.01	0.03
Cadmium	1	<0.01	<0.01	<0.01	<0.01	<0.01	<0.01	NA	NA	<0.01	<0.01
Chromium	1500	6.53	<0.01	4.24	<0.01	4.04	<0.01	3.82	<0.01	4.72	<0.01
Copper	40	5.74	0.14	4.33	0.11	4.06	0.10	5.11	0.13	4.17	0.10
Iron	700	77.1	0.11	73.4	0.11	64.6	0.09	75.4	0.11	81.2	0.12
Lead	3.5	0.04	0.01	0.02	0.01	0.42	0.12	0.55	0.16	0.70	0.20
Nickel	20	3.85	0.20	2.67	0.13	2.68	0.13	2.44	0.12	3.11	0.16
Zinc	300	52.1	0.17	43.9	0.15	24.7	0.08	11.6	0.04	33.4	0.11

*HHI*_*C*_ mean health hazard index for children, *DEI*_*C*_ mean daily element intake from surface soil through corn for children, *RfD*_*0*_ reference dose of the element. DEI_C_ and RfD_0_ in µg/person/day/kg bwt, *NA* not applicable.

## Discussion

We analysed eight trace elements to investigate the contamination status of farmlands in the vicinity of OCP, the largest cement-manufacturing plant in SSA and a reference site. The average surface soil contents of Cd and Pb respectively determined in our work (Table 3) were below the limits (in µg/g) of 50 and 164, respectively set by the National Environmental Standards and Regulation Enforcement Agency (NESREA), Nigeria for surface soil in Nigeria, quoted in Afolayan [30]. The mean Cd concentrations (LOD = 0.04 ng/g) in surface soil from all the farmlands including control (Table 3) were observed to be several orders of magnitude lower than the average contents (in µg/g) of 1.22 and 0.78 (control site) documented from a previous research in the area [31]. However, corn was not investigated and soil samples were taken from sites in the immediate vicinity at 0.5–1 and 3 (control site) km radii of OCP, unlike in our study. In contrast, we found the average Ni and Zn values (Table 3) to be several orders of magnitude higher than the results earlier shown in surface soil (in µg/g ± standard deviation (SD)) for Ni (0.07 ± 0.02) and Zn (0.04 ± 0.01), while the concentrations of Cu (3.42 ± 0.70) and Pb (8.40 ± 2.48) [31] were comparable to our findings (Table 3).

The average levels of Cd, Cr, Cu, Pb and Zn (Table 3) were much lower than average concentrations reported in surface soil in the surrounding areas of cement plants in Nigeria [32] and South Africa [33], except the mean Cr level in the site, north-east of the South African plant. The elevated levels of Pb [32, 33] and Cu [32] were attributed to emissions from the cement plants as well as to the influence of vehicular traffic. Furthermore, the mean As and Ni levels in surface soil in the vicinity of the South African plant [33] were several orders of magnitude higher than what was found in our study. However, the sampling sites were within a 50 m axis of the plant in South Africa [33], while the soil type was Ferralitic and Ferruginous in the sampling sites investigated in Nigeria [32]. Positive correlations were found between Cu and Pb in surface soil in our study: FC (0.521, *p* < 0.05), F1 (0.685, *p* < 0.005), F2 (0.960, *p* < 0.0001), F3 (0.989, *p* < 0.0001) and F4 (0.760, *p* < 0.005), just like previously observed in the surrounding of another cement plant in Nigeria [32]. Similarly, positive associations were shown between As and Pb in surface soil from our work: FC (0.497, *p* < 0.05), F1 (0.578, *p* < 0.01), F2 (0.980, *p* < 0.0001), F3 (0.970, *p* < 0.0001) and F4 (0.658, *p* < 0.05), like that earlier exhibited in the vicinity of the cement plant in South Africa [33]. These findings may indicate the influence of atmospheric depositions and plant cycling which enhances As concentration in surface soil, and together with strong linkages with organic matter and low mobility also concentrate Cu and Pb [9].

Our results were further compared to global parameters owing to the near absent local reference and maximum limit standards in Nigeria. The average Cr levels in surface soils (Table 3) from FC and F1 upwind of OCP were elevated beyond the world soil average [1, 17]. This may be due to the parent material geology from which the soil was formed [34]. The average contents of the other elements determined were below their reference levels. Excluding Cr, the average surface soil concentrations of the trace elements from all the farmlands were below the average levels reported for agricultural soils in Sweden [35] and Japan [36]. In addition, the mean surface soil contents of Cr from F2-F4 downwind of OCP (Table 3) were less than the mean level found in cultivated soil in Japan [36], while the average surface soil Cr level from F3 only was less than what was reported in cultivated soil from Sweden [35]. Regardless, the mean surface soil Cr concentrations from all the farmlands including control were within the maximum allowable concentration of 200 µg/g in soils [1].

The element concentrations with higher RSE values in surface soil (Table 3) than in corn (Table 4) may be due to the heterogeneity of the soil samples and could pinpoint to particle depositions for example. Such findings become manifest when a lot of samples are collected for investigation as was done in our study. Uptake and translocation of elements by plants from the soil is a kinetically controlled process [3]. It is based on the huge differences in the bioavailabilities and chemical reactivities of the different fractions of elements in soil which include the water-soluble, exchangeable, residual fractions and the carbonates, organic matter, and Fe–Mn oxides bounded fractions [19, 37].

Excluding Zn, average element concentrations were lower in corn than in surface soil (Tables 3 and 4). This may be due to a physiological requirement for Zn for optimal growth of the corn plant resulting in the higher uptake from the surface soil. Furthermore, Zn is the only element out of those investigated that has constant mobility in soils [6, 38, 39], and is usually added to fertilisers to improve the yield of corn. The mean corn levels of all the elements determined were lower than the average concentrations reported in corn in an industrial area in Greece [17]. However, the average Cr levels in corn from all the farmlands were several factors greater than the stable concentration range of 0.01–0.41 µg/g reported in cereal grains, although the mean corn contents of Cu, Fe, Ni and Zn from the farmlands were within the range of values for cereals, except the average corn Zn concentrations in F3 and F4 [18]. Our findings could lead to Zn deficiency in corn in F3 and F4. The mean corn Pb concentrations in FC and F1 upwind of OCP were within the range of values found in cereal grains by Eriksson [35]. The average corn Pb contents from F2–F4 downwind of OCP were several orders of magnitude greater than the mean concentrations from FC and F1 upwind of the plant and were above the regulatory limit of 0.2 µg/g set by the Food and Agriculture Organization of the United Nations/World Health Organization (FAO/WHO) [40]. Twelve percent of the corn samples had Pb contents that were a factor of two greater than this limit or higher. Tukey pairwise comparisons revealed statistically significant differences (*p* < 0.0001) in the average Pb and Zn concentrations in *Zea mays* sampled from farmlands upwind of OCP compared to the mean levels in corn sampled from farmlands downwind of the plant.

**Fig. 3:**
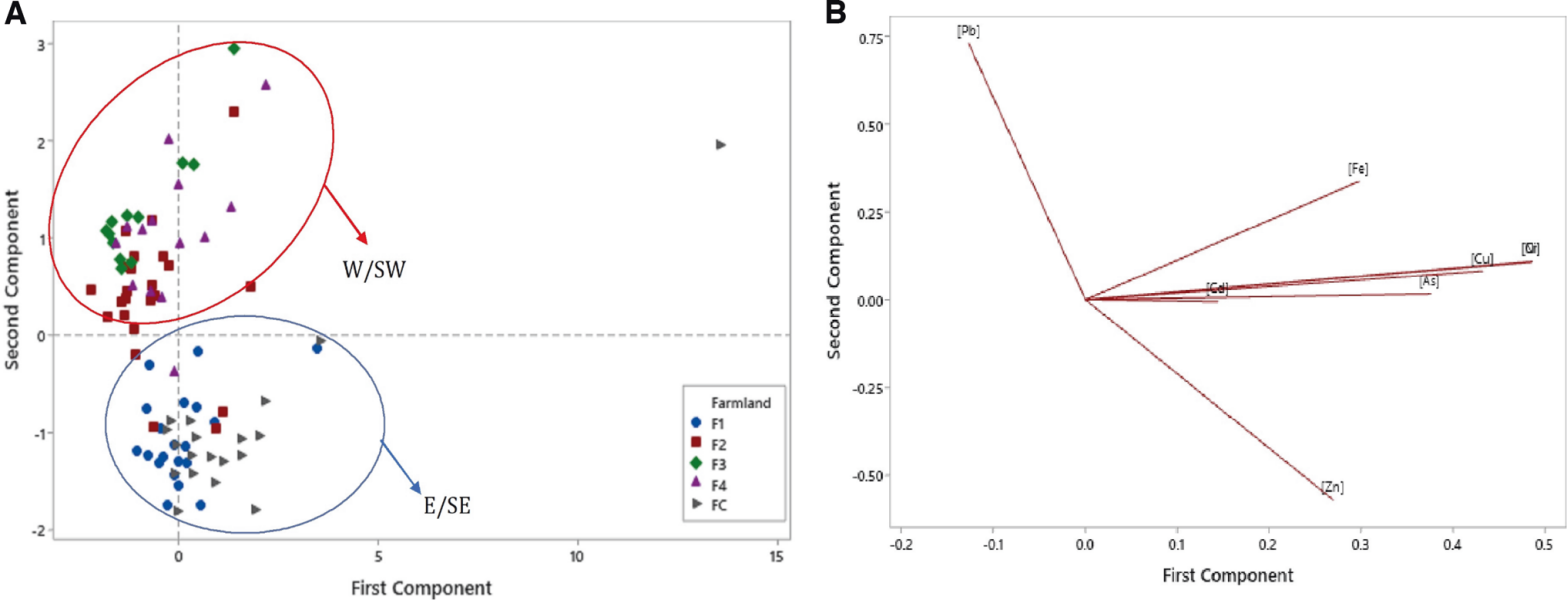
Trace element concentration in corn. **A** Loading plot (**B**) Score plot. W/SW West/South-west, E/SE East/South-east.

**Fig. 4:**
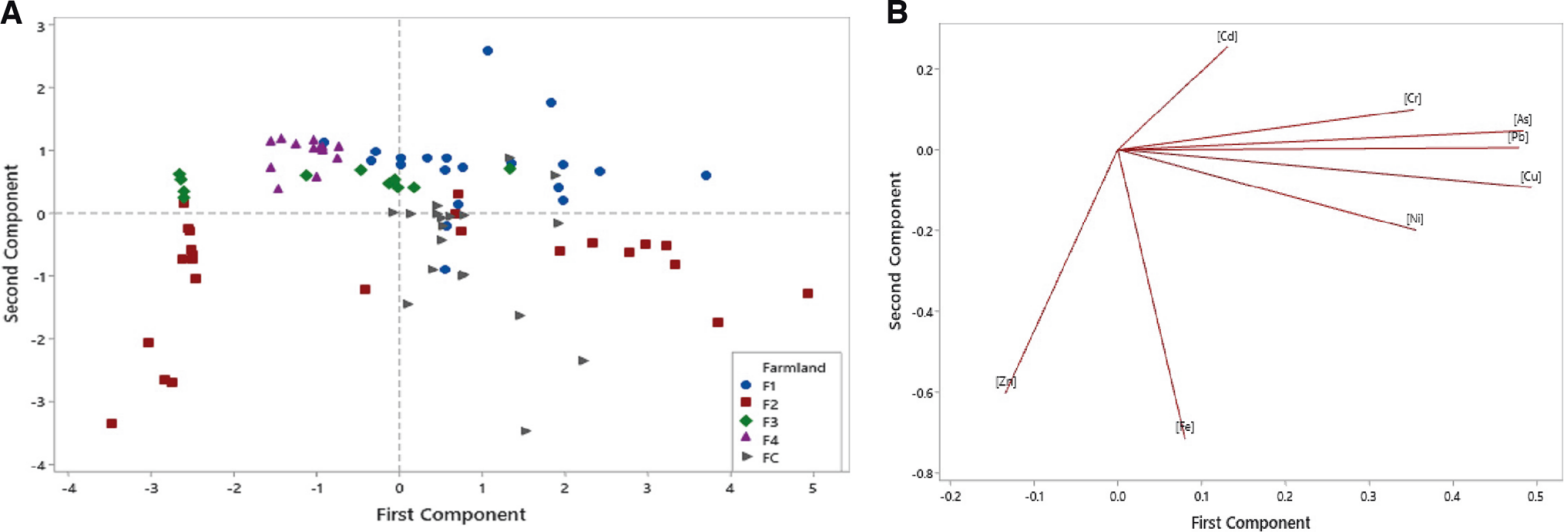
Trace element concentration in surface soil. **A** Loading plot (**B**) Score plot.

These results were confirmed by the PCA score and loading plots for the elements studied in corn (Fig. 3). The score plot (Fig. 3A) showed that the second component was generally effective at separating the data values into those derived from farmlands upwind located east and southeast of OCP or downwind located west and southwest of the plant. The loading plot (Fig. 3B) highlighted Pb and Zn respectively as the greatest contributors to principal component 2 (PC2) loading, with eigenvector values of 0.73 and −0.57, respectively. This implied that while the mean Pb concentrations in *Zea mays* reflect anthropogenic impact in F2–F4 downwind of OCP, the average corn Zn contents did not, and were even found to be higher in corn sampled from FC and F1 (Table 4) which were located upwind of OCP. The PC2 result for the corn Pb contents from farmlands downwind of OCP may have been due to the emission release from the cement stack getting transported in the wind direction mainly (Fig. 3). However, the plume would only occur locally against wind and this was the area sampled upwind of OCP. In contrast, the score and loading plots for the elements’ concentrations in surface soil (Fig. 4) showed that PC1 had a partial impact on separating the data values than PC2. In descending order, the greatest contributors to PC1 loading in surface soil were Cu, As and Pb respectively with eigenvector values of 0.49, 0.48 and 0.48, respectively (Fig. 4).

Arsenic, Cd and Pb are considered as trace elements of environmental concern. They are the only ones among the elements investigated for which their total concentrations are controlled in cereal grains by some organisations. The average DEI_A_/DEI_C_ values for the individual elements from all farmlands are listed in Tables 6 and 7. There are no regulatory limits set by NESREA in Nigeria for trace element content in grains. However, we found the mean DEI_A_/DEI_C_ values for As in corn from all the farmlands were within the regulation limits of 0.5 µg/g in cereal grains established by China [41] and the average DEI_A_/DEI_C_ values for Cd from all the farmlands were within the limits of 0.1 µg/g set by FAO/WHO [40] and China [41]. Furthermore, the average DEI_A_/DEI_C_ values for Pb from F2–F4 downwind of OCP exceeded the limit of 0.2 µg/g set for cereal grains by FAO/WHO [40], 1995) and China [41]. The mean DEI_C_ levels for Pb from the farmlands downwind were two to three factors over this limit. However, the regulatory limit is not a daily intake value and our findings may not necessarily imply that Pb would pose a health hazard through the consumption of corn cultivated in F2-F4 located downwind of OCP. Some studies (e.g., Andersson and Nilsson [42]) had found the status of essential elements like Fe and Zn in food could impact the possible risk of non-essential elements like Pb. Although Fe and Zn intake through corn was of the same order of magnitude from all the farmlands, the real risk due to Pb contamination needs to be investigated in F2–F4 located downwind of OCP which had lower corn Zn contents than in FC and F1 upwind of the plant. (Tables 6 and 7).

An important aspect of human exposure to trace elements via the food chain is the soil-to-plant transfer factor (TF) which may indicate possible human health impact from enriched soils [27]. The TF values for Cd and Zn from the five farmlands including control were wide-ranging and may reflect the low detectability of the elements in most of the surface soil samples. Our sampling regime occurred during the rainy season in the summer of 2017. This result could be due to element mobility in the surface soil which could produce its loss by leaching if excessive rainfall takes place. Other studies have reported such findings for Zn, and Cu has been found to interfere with the adsorption of the metal in soil [43], while precipitation is not the main mechanism of retention of Zn in soils [34]. In addition, the high organic content and clay in the surface soil as discussed in the preceding section might have enhanced Cd sorption [44]. The TF values for As, Cr, Cu, Fe and Ni were of the same order of magnitude in all the farmlands including control. However, TF values for Pb were one to three orders of magnitude higher in F2-F4 downwind of OCP than the values calculated in FC and F1 upwind of the plant, just as found with the Pb concentrations in corn across the farmlands. This may be due to multiple factors such as prevailing wind direction [45], and the density of the local traffic [46]. Other variances in TF values as shown with Cu may be linked with soil characteristics and soil nutrient management [47] which were not investigated in this study. The average TF values for Ni and Zn from all the farmlands were at least two factors lower than what was reported in an earlier research, while the mean TF values for As, Cd, Cu, Fe and Pb from all the farmlands were several orders of magnitude lower than what was documented [17]. However, the TF values for Cr from the two studies are of the same order of magnitude. This could be due to the varying causes of contamination and the differences in the soil signature from the two studies as the soil type in the previous study was well-drained Typic Haploxerepts [17].

The HHI_A_/HHI_C_ values of the trace elements investigated are listed in Tables 6 and 7. All HHI_A_/HHI_C_ values for the investigated elements were less than one (<1) from all the farmlands including control. Our results are in contrast with the findings of an earlier study which showed HHI values greater than one (>1) for all the elements investigated [17] and implies that consumers of corn cultivated in the farmlands in the vicinity of OCP would not experience significant health hazards from the individual trace element. The total sums of individual health hazard indices HHI_A_/HHI_C_ from the farmlands were in descending order F4 (0.39/0.72) > FC (0.38/0.70) > F3 (0.31/0.57) > F2 (0.30/0.56) > F1 (0.30/0.54). Although related health hazards with the elements’ contamination in corn and soils cannot be determined based on the level of exposure alone [27] our data presented here (Tables 6 and 7) implied no risk from the trace elements through the consumption of corn from all the farmlands.

## Conclusions

The health hazards posed by exposure to trace elements As, Cd, Cr, Cu, Fe, Ni, Pb and Zn to communities neighbouring a major cement plant in Obajana, north-central Nigeria through the consumption of corn were investigated based on human health indices (HHIs). The results showed HHI values were less than one for both adults and children for the consumption of corn and also imply that health hazards involving a single trace element are not significant. The trace element concentrations in the surface soil from the farmland chosen as control were higher than the levels in farmlands downwind of the plant and showed the impact of the cement factory dust to be negligible.

## Data Availability

The dataset generated and/or analysed in the current study can be obtained from SA-U on a reasonable request.
